# Protocol for the generation of low-input Hi-C sequencing libraries of FACS-isolated mitotic cells

**DOI:** 10.1016/j.xpro.2025.104241

**Published:** 2025-11-28

**Authors:** Ashley Nichols, Eralda Salataj, Yujin Choi, Pierre-Jacques Hamard, Richard Koche, John Maciejowski

**Affiliations:** 1 Molecular Biology Program, Sloan Kettering Institute, Memorial Sloan Kettering Cancer Center, New York, NY 10065, USA; 2 Center for Epigenetics Research, Memorial Sloan Kettering Cancer Center, New York, NY 10065, USA

**Keywords:** Cell Biology, Cell isolation, Flow Cytometry, Genomics, Sequencing, Molecular Biology

## Abstract

High-throughput chromosome conformation capture (Hi-C) is a powerful tool to investigate 3D genome architecture. Here, we present a protocol for preparing low-input Hi-C libraries from mitotic cells isolated by fluorescence-activated cell sorting (FACS) to study chromatin conformation in mitotic cells. We describe steps for mitotic arrest, harvest and fixation of cultured cells, staining with an anti-Mitotic Protein Marker (MPM2), and isolation of mitotic cells. We then detail procedures for quantifying input material for Hi-C in mitotic cells and library preparation of Hi-C ligated material.

For complete details on the use and execution of this protocol, please refer to Nichols et al.^1^

## Highlights

• Steps for obtaining a pure mitotic cell population via MPM2 staining and FACS sorting
• Instructions for low-input Hi-C using optimized Arima Hi-C kit
• Enables generation of high-quality Hi-C libraries from <1 million mitotic cells

## Abstract (editor highlights)

Publisher’s note: Undertaking any experimental protocol requires adherence to local institutional guidelines for laboratory safety and ethics.

## Graphical abstract

**Figure:**
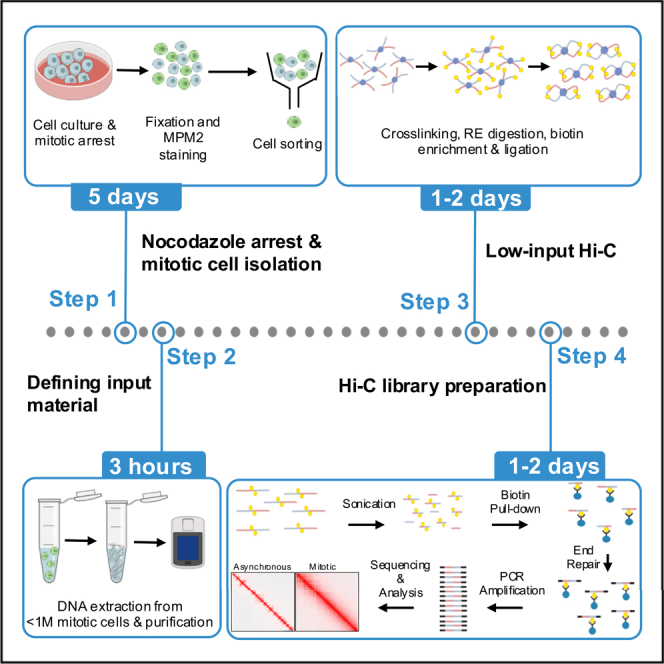

## Teaser

High-throughput chromosome conformation capture (Hi-C) is a powerful tool to investigate 3D genome architecture. Here, we present a protocol for preparing low-input Hi-C libraries from mitotic cells isolated by fluorescence-activated cell sorting (FACS) to study chromatin conformation in mitotic cells. We describe steps for mitotic arrest, harvest and fixation of cultured cells, MPM2 staining, and isolation of mitotic cells. We then detail procedures for quantifying input material for Hi-C in mitotic cells and library preparation of Hi-C ligated material.

## Before you begin

Mitosis is a period of profound structural reorganization, when chromosomes condense, transcription largely ceases, and genome-wide interactions are extensively remodeled.^2^^,^^3^^,^^4^^,^^5^ Many fundamental questions remain about how the condensed mitotic state influences genome architecture and long-range contacts, and whether these changes carry functional consequences for cell division and subsequent interphase organization. Hi-C sequencing enables the study of three-dimensional (3D) genome organization, but most published protocols require large input cell numbers, which can be challenging when working with rare populations such as mitotic cells.^6^ Isolating a pure mitotic population is essential to address these questions and prevent background asynchronous signal in downstream Hi-C sequencing analyses, yet doing so in a cost- and time-efficient manner has remained a barrier, especially for slow-growing or difficult-to-synchronize lines. The traditional method of mitotic shake-off can lead to contamination of mitotic samples with asynchronously growing cells, complicating downstream Hi-C analyses.^7^ An ideal approach overcomes these challenges by enabling high-purity mitotic isolation from relatively small cultures, broadening access to mitotic Hi-C for studies of chromosome dynamics, genome stability, and cell cycle regulation.

### Innovation

Here, we describe a fluorescence-activated cell sorting (FACS)-based approach to isolate mitotic cells with high purity using an anti-Mitotic Protein Marker (MPM2) antibody, which recognizes phosphorylated LTPLK motifs characteristic of mitotic proteins.^8^ We pair this isolation strategy with optimized Arima Hi-C from as few as 300,000 cells, reducing both reagent cost and the need for large-scale cultures. We have made alterations to the Arima workflow, including changes in cell numbers, fixation, extended lysis time, and reduced library amplification cycles. This low-input workflow allows researchers to perform mitotic Hi-C even when starting material is limited, facilitating studies in a time-efficient and cost-effective manner. Here, we present a protocol for the generation of mitotic Hi-C libraries from as few as 300,000 nocodazole-arrested COLO320-DM cells. However, we obtained similar results using COLO320-HSR and HCT116 cells.

For low-input Hi-C, we highly recommend setting up a small-scale sorting experiment before executing this protocol in its entirety. This will help define the sorter conditions and evaluate the amount of cells needed to obtain the minimum input material (i.e., determine how much genomic DNA is obtained from 300,000 to 500,000 sorted, mitotic cells). To test the feasibility of low-input Hi-C on your cell line, we recommend completing Steps 1–2 (Cell Harvest and Mitotic Cell Isolation, MPM2 staining and isolation of mitotic cells) and Step 3 (Defining Input Material for Hi-C in Mitotic Cells) of this protocol before designing larger-scale experiments and proceeding with Hi-C.

Please reference the Arima Hi-C User Guide for additional information and successful use of this protocol.

## Key resources table

**Table:** 

REAGENT or RESOURCE	SOURCE	IDENTIFIER
**Antibodies**		

MPM2	Abcam	Cat#ab14581; RRID: AB_301354
Goat anti-mouse IgG Alexa Fluor 488	Invitrogen	Cat#A-11001; RRID: AB_2534069

**Chemicals, peptides, and recombinant proteins**		

Ethanol	Fisher Scientific	BP2818-500
Fisher BioReagents bovine serum albumin, fraction V, cold-ethanol precipitated	Fisher Scientific	Cat#BP1605-100
Nocodazole	Cayman	Cat#13857-50
Protease inhibitor tablets, EDTA-free	Thermo Scientific	Cat#A32965
Paraformaldehyde	Fisher Scientific	Cat# AC416785000
SPRI magnetic beads	Beckman Coulter	B23319
DAPI	Fisher Scientific	P36962
Propidium iodide (PI)	DeNovix	CD-PI-1.5
1× PBS	Fisher Scientific	MT21040CV
Proteinase K	New England Biolabs	Cat# P8107S
GlycoBlue coprecipitant (15 mg/mL)	Thermo Fisher Scientific	Cat# AM9516
Glycine	Fisher Scientific	Cat# BP381-500
Poly L-lysine solution	MilliporeSigma	Cat# P4707-50ML

**Critical commercial assays**		

Arima Hi-C library preparation kit	Arima	Cat# A303011
Arima Hi-C kit (8 reactions)	Arima	Cat# A510008
Qubit 1× dsDNA high sensitivity (HS) and broad range (BR) assay kits	Thermo Fisher Scientific	Cat# Q33266
RPMI cell culture media	MSKCC Media Core Facility	N/A
Fetal bovine serum	MSKCC Media Core Facility	N/A
Penicillin/streptomycin (100×)	Thermo Fisher Scientific	Cat# 10378016
Trypsin 0.05% EDTA 0.02% in Hanks’ balanced salt solution (HBSS) with phenol red and without calcium/magnesium	MSKCC Media Core Facility	N/A

**Experimental models: Cell lines**		

COLO320-DM	ATCC	Cat #CCL-20; RRID:CVCL_0219

**Software and algorithms**		

ImageJ	Schindelin et al.^9^	https://imagej.net/software/fiji/
Hi-C Pro	Servant et al.^10^	https://nservant.github.io/HiC-Pro/

**Other**		

JS-5.3 swinging bucket rotor	Beckman Coulter	Cat# 368690
FACSAria III cell sorter	BD Biosciences	N/A
PCR thermocycler	Applied Biosystems	Cat# 4483636
Rotator	Thermo Fisher Scientific	Cat# 88881001
Magnetic rack	EpiCypher	Cat# SKU-10-0008
Sonicator	Covaris	Cat# E220
Covaris 130 μL tubes	Covaris	Cat# 520045
TapeStation	Agilent	Cat# 4200
HS D5000 buffer + ladder (Agilent)	Agilent	Cat# 5067-5589
HS D5000 tapes (Agilent)	Agilent	Cat# 5067-5588
Microscope cover glass	Fisher Scientific	Cat# 50-143-783
Eppendorf DNA LoBind tubes	Fisher Scientific	Cat# 13-698-791
0.2 mL PCR tubes	Eppendorf	Cat# 2231001160
Corning TC-treated culture dishes (150 mm)	Fisher Scientific	Cat# 08-772-24
Fisherbrand Easy Reader conical polypropylene centrifuge tubes (50-mL)	Fisher Scientific	Cat# 05-539-13
Fisherbrand Easy Reader conical polypropylene centrifuge tubes (15-mL)	Fisher Scientific	Cat# 07-200-886
5 mL serological pipette	Fisher Scientific	Cat # 13-675-22
10 mL serological pipette	Fisher Scientific	Cat # 13-675-20
25 mL serological pipette	Fisher Scientific	Cat # 13-675-30
Falcon 5-mL round-bottom tube with a strainer cap	Fisher Scientific	Cat # 08-771-23
Eppendorf ThermoMixer C	Fisher Scientific	Cat# 05-412-503
Qubit 3.0 fluorometer	Thermo Fisher Scientific	Cat# Q33216
Qubit tubes	Thermo Fisher Scientific	Cat# Q32856
Nikon Eclipse Ti2-E CSU W-1 spinning disk	Nikon	N/A

## Materials and equipment

### Sorting wash buffer

30% BSA diluted in 1× PBS to a final concentration of 0.2%. Wash buffer can be stored at 4°C for 1 week.

### Sorting blocking buffer

30% BSA diluted in 1× PBS to a final concentration of 1%. Blocking buffer can be stored at 4°C for 1 week.

### 2.5 M glycine

Dissolve 9.384 g of glycine in 40 mL of Milli-Q water. Adjust the final volume to 50 mL. Sterilize with a 0.22-μm filter and store at 20°C–25°C for several months.

### 2% PFA

Add 10 g of paraformaldehyde (PFA) to 100 mL ddH_2_O. Add 200 μL 2 N NaOH. Stir mixture on hot plate at 65°C until PFA is dissolved. Once the solution is clear, add 50 mL 10× PBS and 350 mL ddH_2_O. Stir mixture and check pH. pH of 2% PFA should be 7.2–7.3. If pH is too high, adjust pH by adding dilute HCl dropwise. Store in 10 mL aliquots at −20°C for several months.

**CRITICAL:** PFA is highly toxic and should be prepared in a chemical hood. NaOH and HCl are corrosive. PFA should always be handled in a fume hood. Wear laboratory coat, gloves and goggles when working with PFA, NaOH and HCl.

**Table:** Permeabilization buffer

Reagent	Final concentration	Amount
1 M Tris HCl pH 8.0	10 mM	2 mL
1 M NaCl	10 mM	2 mL
20% IGEPAL CA-630	0.2%	2 mL
EDTA Protease Inhibitor Cocktail	N/A	4 tablets
PBS	N/A	194 mL
**Total**	**N/A**	**200 mL**

∗Prepare fresh before each use and store at 4^o^C until needed.

***Note:*** We have not tested effectiveness of permeabilization when the buffer is not prepared fresh before use.

**Table:** Sorting Buffer

Reagent	Final concentration	Amount
30% BSA	1%	0.3 mL
20 mg/mL RNase A	0.2 mg/mL	100 μL
PBS	N/A	9.57 mL
**Total**	**N/A**	**10 mL**

∗Prepare fresh before each use and store at 4°C until needed.

**Table:** 2× Decrosslinking Buffer

Reagent	Final concentration	Amount
1 M Tris HCl, pH 8.0	100 mM	1 mL
0.5 M EDTA	2 mM	0.04 mL
10% SDS	2%	2 mL
5 M NaCl	400 mM	0.8 mL
ddH2O	N/A	6.16 mL
**Total**	**N/A**	**10 mL**

∗Store at 20°C–25°C for up to 6 months.

***Alternatives:*** In this protocol, COLO320-DM cells were arrested in prometaphase via a nocodazole arrest.^11^ However, different cell-cycle inhibitors may be utilized to isolate cells at different stages of mitosis. Additionally, the efficacy of the nocodazole arrest may vary between cell lines. Other mitotic arrest agents or nocodazole treatment optimization may be needed to obtain a sufficient number of mitotic cells for Hi-C library preparation. To circumvent the need for staining cells with an anti-MPM2 antibody, other options to isolate mitotic cells include generating cell lines expressing fluorescently labeled proteins that are highly expressed during mitosis, including Plk1 or Cyclin-B1.^12^^,^^13^

### Flow cytometry sorting setup

**Timing: 30 min**

All sorting experiments were established with the BD FACSAria III Cell Sorter with the 130 μm nozzle. To obtain the purest population, sorting was conducted using the four-way purity mode. The sorter needs to have a refrigerated collection tube holder to keep sorted samples cold during the sorting process.

***Alternatives:*** Other cell sorters are likely suitable for this application but have not been tested.

***Note:*** Use of the 130 μm nozzle ensures a mild stream to prevent extensive damage to cells during the sort. Smaller nozzles will lead to greater pressure on the cells and their nuclei during sorting and potentially disrupt 3D chromatin interactions.

Perform quality controls and Accudrop, and select the desired filtered sets.

• GFP channel: Laser 488–530/30.

***Alternatives:*** Lasers can be altered if the secondary fluorescent antibody is a different fluorophore (ex: Alexa Fluor 647). Select the excitation laser whose wavelength most closely matches the fluorophore’s excitation maximum.

Adjust the voltages for each channel, including forward-scatter (FSC), side-scatter (SSC), and GFP channels, to ensure that the positive populations are present on the displayed scale, using the following controls (GFP channel should be in logarithmic view for better visualization of the two GFP+ populations).

• Unstained cells.
• Asynchronous cells not treated with nocodazole and fully stained with anti-MPM2 primary and GFP secondary antibody.
• Nocodazole-treated cells fully stained with anti-MPM2 primary and GFP secondary antibody.

***Note:*** For COLO320-DM mitotic cell sorting, typical voltage thresholds are: FSC, 410 V; SSC, 210, and GFP, 200.

First, use the unstained cells to set the voltages of FSC, SSC, and GFP. Then, use the fully stained asynchronous cells to set the voltage of the GFP channel.

**Figure 1:**
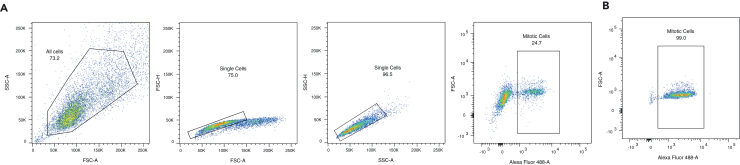
Sorting strategy for FACS-isolation of MPM2-labeled mitotic cells (A) Gating strategy for isolation of mitotic or asynchronous cells from MPM2-labeled COLO320-DM cells following a four-hour nocodazole arrest. (B) Post-sort analysis of sorted mitotic cells to determine sort purity. Samples were rerun using the same gates as (A) to determine sort purity.

Set the gates to isolate the mitotic cell population (see Figure 1).

• Display all events in a graph [x-axis = FSC-A – y-axis = SSC-A] (see Figure 1A).
• Draw gate 1 around the population of all cells, removing debris.
• Display gate 1 in a graph [x-axis = FSC-A – y-axis = FSC-H] (see Figure 1A).
• Draw gate 2 [x-axis = FSC-A – y-axis = FSC-H] and gate 3 [x-axis = SSC-A – y-axis = SSC-H] around the populations of single cells to remove doublets.
• Draw gate 4 around the population of mitotic cells (i.e., with high GFP signal intensity). Enrichment of high-GFP cells should be seen between asynchronous and nocodazole-arrested cells.

**CRITICAL:** The samples should never be vortexed. Vortexing the sample can lead to irreversible damage of the 3D structure of the nuclei, which can compromise the outcome of the experiment.

***Note:*** To homogenize the samples, gently invert the FACS tubes 5 times. Pause sorting throughout the experiment to homogenize the sample if cells begin to sediment at the bottom of the tube.

## Step-by-step method details

### Mitotic arrest, harvest, and fixation of cultured cells

**Timing: 5 days**

This major step describes the process for culturing and fixing mitotically arrested cells using 2% PFA. The number of cells seeded here yielded between 300,000 to 1 million FACS-isolated mitotic cells.

1. Seed 5 × 10^6^ cells in eight 15-cm cell culture dishes.
  a. Plate 2 × 10^6^ in a 10-cm culture dish to be used as an unstained control during sorting.
  b. Plate 2 × 10^6^ in a 10-cm culture dish to be used as an asynchronous, stained control during sorting.

***Note:*** Based on the efficacy of the nocodazole arrest in your chosen cell line, the number of 15-cm plates can be scaled up or down.

2. Culture cells until they reach ∼70% confluency.
3. Treat cells in 15-cm dishes with 100 ng/mL nocodazole for 4 h.

***Note:*** This treatment regimen can be optimized and modified depending on cell line.

4. Harvest and fix cells.
  a. Remove cell culture media and wash once with sterile 1× PBS. Reserve culture media in a 50 mL conical tube.
  b. Cover cells with ∼5 mL trypsin-EDTA and place at 37°C until cells have detached.
  c. Quench trypsin with reserved culture media. Spin down cells at 300 × *g* for 5 min.
  d. Wash the cell pellet once with 1× PBS. Spin cells down at 300 × *g* for 5 min.
  e. Resuspend the cell pellet in 15 mL of 2% PFA for 15 min at 20°C–25°C. Invert the tube periodically to keep cells suspended and prevent settling at the bottom of the tube.
  f. Quench the reaction by adding 700 μL 2.5 M glycine to each tube (final concentration of glycine should be 125 mM). Incubate for 5 min and invert periodically to prevent settling.
  g. Spin down cells at 300 × *g* for 5 min and aspirate PFA. Wash pellet twice with 1× PBS.
  h. Resuspend pellet in 10 mL 1× PBS and place at 4°C for 12–16 h.

***Note:*** This protocol was optimized and tested on cells stored 12–16 h at 4°C. Longer storage times may result in nucleic acid degradation, but have not been tested.

### MPM2 staining and isolation of mitotic cells

**Timing: 10 h**

This major step describes a protocol to stain mitotic cells with anti-MPM2 primary antibody and goat anti-mouse GFP secondary antibody. The stained cells are then sorted with a FACSIII Aria Cell Sorter to obtain a mitotic population with about a 99% purity. Because staining and sorting are a time-consuming process, we recommend collecting the sorted samples, then snap-freezing and storing at −80°C before starting Hi-C on a different day.

**Figure 2:**
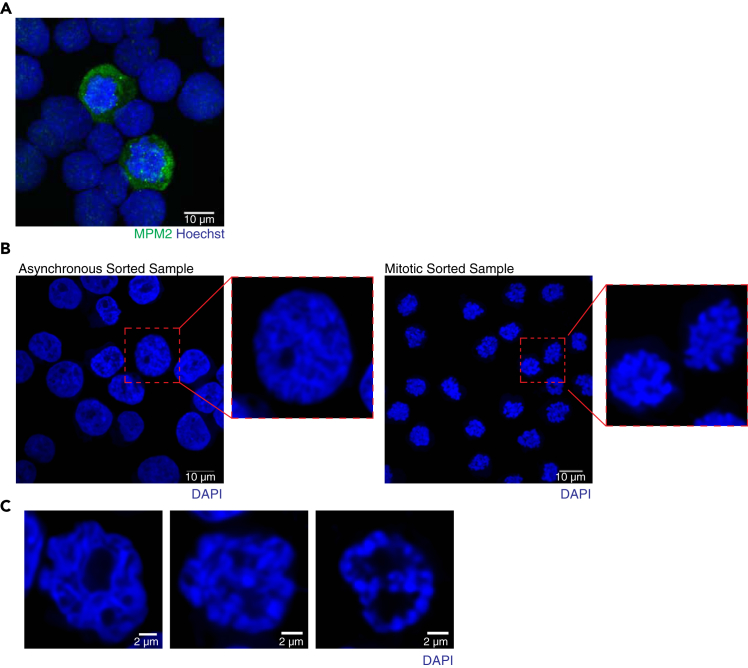
Isolation of MPM2-labeled mitotic cells preserves 3D chromatin interactions (A) anti-MPM2 immunofluorescence (IF) of COLO320-DM cells treated with nocodazole for four hours. Maximum intensity projection depicted showing MPM2 labeling (green) in mitotic COLO320-DM nuclei (blue). Scale bars = 10 μm. (B) Representative single z-stack images portraying COLO320-DM cells post-sorting. Following sorting, asynchronous (left) and mitotic (right) were spun onto coverslips and stained with DAPI. Insets depicts healthy cell nuclei with intact 3D structure as shown by smooth perinuclear edges by DAPI staining (left) and mitotic cell nuclei in mitotic sorted sample (right). Scale bars = 10 μm. (C) Examples of asynchronously sorted cells with disrupted 3D structure. Scale bars = 2 μm.

5. Stain cells with anti-MPM2 antibody.
  ***Note:*** Fixed cells from the untreated 10-cm dish should be reserved to be used as an unstained negative control during sorting.
  ***Note:*** Asynchronous cells to be used as a control undergo the same staining and sorting protocol.
  ***Note:*** All buffers and centrifugation spins should be kept at 4°C. Samples should always be kept on ice unless otherwise noted.
  a. Spin cells down at 400 × *g* for 5 min at 4°C. Aspirate PBS.
  b. Permeabilize cells for 30 min using pre-made, cold permeabilization buffer on ice.
  c. Spin cells down at 400 × *g* for 5 min at 4°C and aspirate permeabilization buffer.
  d. Wash the cell pellet twice in 0.2% BSA wash buffer. Spin cells down at 400 × *g* for 5 min at 4°C in between each wash.
  e. Block cells in blocking buffer (1% BSA) for 1 h on ice. Invert cells periodically to keep cells suspended and prevent settling at the bottom of the tube.
  f. After 1 h, spin down cells at 400 × *g* for 5 min. Aspirate supernatant.
  g. Prepare staining buffer (3 mL per 15 mL tube of cells) by diluting anti-MPM2 antibody 1:500 in blocking buffer. See Figure 2A.
  h. Resuspend cells in 3 mL staining buffer for 1.5 h at 20°C–25°C. Place cells on a shaker so they are gently agitated during incubation and do not settle at the bottom of the tube.
  i. After incubation in primary antibody, fill a 15-mL tube with 0.2% BSA solution and spin down at 400 × *g* for 5 min at 4°C.
  j. Wash cells twice in 0.2% BSA wash buffer. Spin cells down at 400 × *g* for 5 min at 4°C in between each wash.
  k. Stain cells with Goat anti-Mouse Alexa Fluor 488 secondary antibody diluted 1:1000 in blocking buffer. Incubate cells in 3 mL of secondary antibody staining solution for 1 h at 20°C–25°C.
    ***Note:*** Keep cells in the dark during incubation.
  l. Fill tube with 0.2% BSA wash buffer and spin down at 400 × *g* for 5 min at 4°C. Aspirate supernatant.
  m. Wash twice with 0.2% BSA wash buffer.
  n. Resuspend in 2.5 mL sorting buffer.
    ***Note:*** Divide sample amongst flow tubes if necessary and dilute further with sorting buffer if sample is too concentrated.
    ***Note:*** While sorting, the flow rate should be 1,500–3,000 events/second. Higher flow rates may lead to excessive stress on cells during the sorting process and decrease the sorting purity. If the sample is too concentrated, the flow rate will be higher so dilute cells further.
6. Sort cells using a BD FACSAria III Cell Sorter (Flow Cytometry Sorting Setup).
  a. Use the unstained control samples to draw the gates for mitotic cells (See Figure 1).
  b. Wash the system by running water between each sample for 1 min at maximum flow rate.
  c. Add ice-cold 2 mL 1% BSA to the collection tube before collecting the sample. Invert the tubes to coat the interior the tubes with the buffer.
    ***Note:*** We sorted into 15-mL conical tubes for downstream Hi-C. To test sort purity, we also used 5-mL FACS-tubes as collection tubes.
  d. Invert the FACS-tube 5 times before beginning sorting to homogenize the sample.
    **CRITICAL:** Do not vortex sample. Avoid vortexing to prevent irreversible damage to cell and 3D structure.
  e. Sort mitotic cells at a flow rate of 1,500–3,000 events/second and 4-way purity mode.
    ***Note:*** Sorting using the 130 μm nozzle and 4-way purity mode is slower than other nozzles/modes. However, this ensures the least amount of stress to cells during the sorting process and sorts cells with the fewest error than other modes. See Figure 2.
  f. Sort ∼10,000 cells into a 5-mL FACS-tube to be reserved for testing sort purity at the end of the sort.
  g. Sort the remainder of the sample into a 15-mL collection tube. Keep sorted samples on ice until finishing the sort (See Figure 1B).
  h. After sorting all samples, load the reserved 5-mL FACS-tube and re-run the sample to test sorting purity.
    ***Note:*** With these conditions, we obtained 99%–100% purity of mitotic cells. Lower purity may occur if faster flow-rates or different conditions are used. See Figures 2A and 2B.
    ***Note:*** Samples should always be kept on ice following sort.
7. After sorting, spin sorted cells down at 300 × *g* for 10 min at 4°C.

***Note:*** If you do not see a cell pellet, or see a very small cell pellet, higher centrifugation speeds can be used. All spins should be performed in a swinging bucket centrifuge. We have successfully used speeds of 2500 × *g*–3000 × *g* for small pellets. However, speeds more than 3000 × *g* might result in cell lysis and compromise the Hi-C experiment.

8. Carefully remove supernatant with a serological pipette to avoid loss of cell pellet.

***Note:*** To avoid loss of cell pellet, avoid aspirating supernatant with vacuum. Gentle removal of the supernatant with a pipet may prevent dislodging the sorted cell pellet. The supernatant can be reserved and spun down again if the cell pellet is not visible.

9. Resuspend pellet in 1-mL 1% BSA and transfer to a DNA LoBind 1.5 mL Eppendorf tube.

**CRITICAL:** It is critical to use low-binding tubes to prevent sample loss due to sticking to sides of the tube.

10. Count cells and determine the total cell number upon sorting.

***Note:*** Sample may be lost due to the sorting process, spins and mechanical stress. Therefore, it is critical to recount samples following sorting to confirm the total number of cells collected.

11. Aliquot samples in batches of 300,000–500,000 cells based on total numbers from sorting.

**CRITICAL:** We have never tested batches below 300K mitotic cells. For such a low number of mitotic, the Hi-C protocol might need further optimization.

12. Spin down cells at 300 × *g* for 10 min at 4°C.
13. Remove supernatant carefully, without disturbing the cell pellet.

**CRITICAL:** All PBS/BSA must be removed to avoid excessive crystal formation during freezing. Crystal formation in cell pellet due to residual PBS will disrupt nuclei and lower DNA quality during Hi-C.

14. Snap freeze pellets using liquid nitrogen.
15. Store cell pellets at −80°C until ready to conduct library preparation.

***Note:*** If cell pellets are not visible, 30% BSA can be added prior to spinning to assist with cell pelleting.

**Pause point:** Cell pellets can be stored at −80°C for up to three weeks before thawing and extracting genomic DNA. Samples can likely be stored at −80°C for months or longer, but we have not tested longer time periods.

### Quantifying input material for Hi-C in mitotic cells

**Timing: 3 h**

This protocol is optimized for low-input samples (fewer than 1 million mammalian cells) using the Arima Hi-C kit. Estimating the input material is important to calculate the amount of chromatin that is needed for the Hi-C. Normally 275 ng-3 μg of chromatin is considered as an adequate material to work with. However, in our lab we have had successful libraries with as low as 150 ng starting material.

16. Thaw one aliquot of sorted, frozen cells (e.g., 300,000 cells) and add 230 μL of 2× Decrosslinking buffer + 1 μl of proteinase K.
17. Incubate at 78°C for 15 min.
18. Incubate at 25°C for 10 min.
19. Add 150 μL of DNA Purification Beads, mix thoroughly, and incubate at RT for 5 min.
20. Place sample against Epicypher magnet and incubate until solution is clear.
21. Discard supernatant. While sample is still against magnet, add 400 μL of 80% ethanol, and incubate at RT for 1 min.
22. Discard supernatant. While sample is still against magnet, add 400 μL of 80% ethanol, and incubate at RT for 1 min.
23. Discard supernatant. While sample is still against magnet, incubate beads at RT for 3–5 min. Avoid over-drying the beads.
24. Remove sample from magnet, resuspend beads thoroughly in 100 μL of Elution Buffer, and incubate at RT for 5 min.
25. Place sample against magnet, incubate until solution is clear, and transfer supernatant to a new tube.
26. Quantify sample using Qubit. The total DNA yield corresponds to the amount of DNA obtained from 300,000 mitotic cells.

***Note:*** For low-input Hi-C we recommend using 200 ng minimum as starting material. If lower than 200 ng than we strongly recommend to bank mitotic cells. Input material that gives less than 200 ng will compromise your library efficiency.

### Hi-C using Arima Hi-C kit

**Timing: 1–2 days**

Hi-C is a genome-wide assay that captures both the sequence and 3D structure of the genome. By using the Arima Hi-C kit, cells are initially crosslinked to preserve genomic interactions. Following lysis, the crosslinked chromatin is further digested with a restriction enzyme cocktail (usually DpnII/NlaIII), and the resulting 5′ overhangs are filled in with biotinylated nucleotides. Proximity ligation joins spatially adjacent DNA ends, capturing 3D genome contacts. Ultimately, the ligated DNA is purified, fragmented, and enriched for biotin-labeled fragments. These are used for library preparation with supported commercial kits, each accompanied by an Arima-Hi-C Library Prep User Guide tailored to the chosen kit. The Arima protocol below has been slightly modified for low-input samples.

27. Thaw the 2% PFA crosslinked cells and resuspend them in 1 ml of 1× PBS/1% PFA containing 3% BSA.
28. Incubate the fixed cells for 10 min at 4^o^C under agitation. Cells should not settle.
29. Add 91.9 μL of Stop Solution 1, mix well by inverting 10 times and incubate at 4°C for 15 min, under agitation.
30. Pellet cells by centrifugation at 500 × *g* for 5 min at 4°C.
31. Discard supernatant carefully without disrupting the cell pellet.
32. Wash cells by adding 1 mL of prechilled 1× PBS containing 3% BSA and resuspend gently.

***Note:*** Some cell types might be more sensitive to the freezing process, so we recommend that at this point you recount the cells by using a PI staining dye.

33. Pellet cells by centrifugation at 500 × *g* for 5 min at 4°C.
34. Discard supernatant, leaving only the crosslinked cell pellet and no residual liquid.
35. Resuspend one aliquot of crosslinked cells in 20 μL of Lysis Buffer in a tube or a well of a PCR plate, and incubate at 4°C for 20 min.
36. Add 24 μL of Conditioning Solution, mix gently by pipetting, and incubate at 62°C for 10 min.
37. Add 20 μL of Stop Solution 2, mix gently by pipetting, and incubate at 37°C for 15 min.
38. Add 12 μL of a master mix containing the following reagents:

***Note:*** When preparing master mixes for the Arima Hi-C protocol, adjust each reagent volume according to the number of reactions required, adding an additional 10% to compensate for potential pipetting losses.

**Table:** 

Reagent	Volume per reaction (μL)
Buffer A	7
Enzyme A1	1
Enzyme A2	4
**Total**	**12**

39. Mix gently by pipetting and incubate under mild agitation (350 rpm) as follows:

**Table:** 

Temperature	Time
37°C	60 min
65^o^C	25 min
25^o^C	10 min

40. Add 16 μL of a master mix containing the following reagents:

**Table:** 

Reagent	Volume per reaction (μL)
Buffer B	12
Enzyme B	4
**Total**	**16**

41. Mix gently by pipetting, and incubate at 20°C–25°C for 45 min under mild agitation (250 rpm).

***Note:*** Avoid making any bubbles while mixing as this will affect the enzymatic digestion.

42. Add 82 μL of a master mix containing the following reagents:

**Table:** 

Reagent	Volume per reaction (μL)
Buffer C	70
Enzyme C	12
**Total**	**82**

43. Mix gently by pipetting, and incubate at RT for 15 min.
44. Add 35.5 μL of a master mix containing the following reagents:

**Table:** 

Reagent	Volume per reaction (μL)
Buffer D	10.5
Enzyme D	25
**Total**	**35.5**

***Note:*** Enzyme D should be warmed to RT to prevent precipitation in the master mix below.

45. Add 20 μL of Buffer E, mix gently by pipetting, and incubate under mild agitation (400 rpm) as follows:

**Table:** 

Temperature	Time
55^o^C	30 min
68°C	90 min
25°C	10 min

46. DNA purification:
  a. Add 100 μL of DNA SPRI Purification Beads, mix thoroughly, and incubate at RT for 5 min.
  b. Place sample against magnet and incubate until solution is clear.
  c. Discard supernatant. While sample is still against magnet, add 200 μL of 80% ethanol, and incubate at RT for 1 min.
  d. Discard supernatant. While sample is still against magnet, add 200 μL of 80% ethanol, and incubate at RT for 1 min.
  e. Discard supernatant. While sample is still against magnet, incubate beads at RT for 3 – 5 min to air-dry the beads.
  f. Remove sample from magnet, resuspend beads thoroughly in 20 μL of Elution Buffer, and incubate at RT for 5 min.
  g. Place sample against magnet, incubate until solution is clear, and transfer supernatant to a new tube.
  h. Quantify sample using Qubit.

***Note:*** If the proximally ligated DNA yield is less than 275 ng, we recommend skipping the Arima-QC1 assay and strongly recommend performing the Arima-QC2 assay described in the Arima-HiC Library Preparation user guide for low-input samples. In any case, if the material recovered is less than 100 ng, we recommend not proceeding with the library prep. At this point, we also recommend pooling material derived from different batches. If the starting material is limited, then we recommend eluting (step 46.f) in a smaller volume so you can bank up proximally-ligated material from different batches.

### Library preparation of Hi-C ligated material

**Timing: 1–2 days**

The Arima Hi-C library preparation process begins with fragmentation of the DNA, followed by size selection and enrichment for biotin-labeled fragments. Next steps include the end-repair, dA-tailing, and adapter ligation, performed while the ligated DNA remains attached to T1 magnetic beads. The final step involves a PCR amplification of the bead-bound DNA using index primers and generates the final Arima-Hi-C library, ready for sequencing. For low-input libraries, we recommend a shallow sequencing of 100M reads as a QC in a PE100 or PE150 mode. After the QC analysis, we recommend a total of 600M reads when aiming for high-resolution analyses of chromatin loops. For additional information on the following steps, please reference the Arima Library Preparation User Guide.

**Figure 3:**
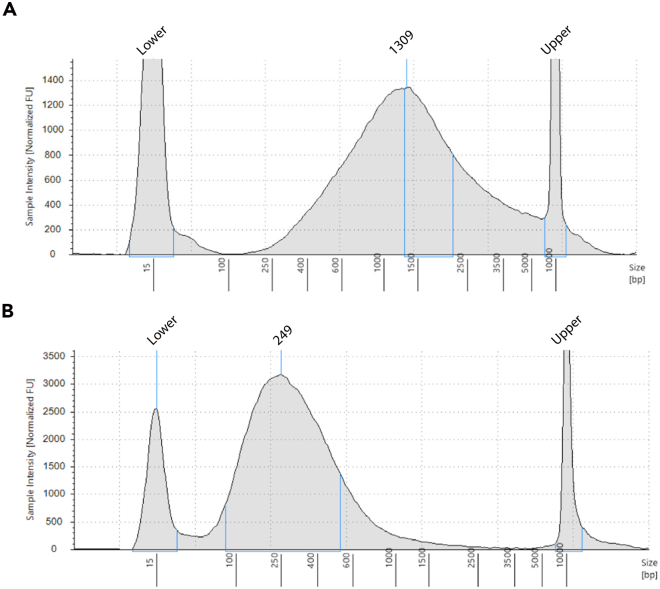
Fragment analyzer traces of proximally ligated Hi-C on mitotic cells (A) Electropherogram that displays fluorescence over migration time, allowing accurate determination of the fragmentation profile using a D5000 screen tape. An optimal proximally ligated Hi-C on mitotic cells shows a smooth profile with a distribution within the range of 250 - 5000 bp. (B) Fragmentation profile of the above ligated material upon sonication for 60 sec in a Covaris instrument. The region encompassing the majority of the proximally ligated material is usually distributed between 100 – 800 bp with a peak around 250 bp. The lowermost (15 bp) and the uppermost (10,000 bp) bands represent the lower and upper DNA markers, respectively.

47. DNA Fragmentation.
  a. If necessary, add Elution Buffer to bring the sample volume to 100 μL. Do not exceed 100 μL of volume for DNA fragmentation.
    ***Note:*** To identify the amount of sonication time, we recommend running the ligated samples on a Tapestation. (Figure 3A).
  b. Fragment DNA to obtain an average fragment size of 200–400 bp. In our hands, Covaris E220 has been performing extremely well for low-input Hi-C.

**Table:** Suggested Covaris E220 Parameters

Parameter	Covaris E220
Peak Incident Power (w)	175 W
Duty cycle/duty factor	10%
Intensity	5
Cycles per burst	200
Treatment time (s)	∼50–80 seconds

    ***Note:*** These settings can be customized based on your actual protocol or instrument settings, if available.
  c. Run an aliquot of fragmented DNA on TapeStation to confirm an appropriate fragment size distribution centered 250–350 bp (Figure 3B).
    ***Note:*** Samples may be stored at −20°C for up to 3 days.
48. DNA size selection.
  Fragmented DNA must be size-selected to have a size distribution between 200–600 bp.
  a. Transfer fragmented DNA sample from fragmentation tube to either a microfuge tube, PCR tube, or PCR plate. If necessary, add Elution Buffer to bring sample volume to 100 μL.
  b. Add 60 μL of DNA Purification Beads, mix thoroughly by pipetting, and incubate at RT for 5 min.
  c. Place sample against magnet and incubate until solution is clear.
  d. Transfer ∼160 μL of supernatant to a new sample tube or well of a PCR plate. Discard beads.
  e. Add 40 μL of DNA Purification Beads to the ∼160 μL of supernatant, mix thoroughly by pipetting, and incubate at RT for 5 min.
  f. Place sample against magnet and incubate until solution is clear.
  g. Discard supernatant. While sample is still against magnet, add 200 μL of 80% ethanol, and incubate at RT for 1 min.
  h. Discard supernatant. While sample is still against magnet, add 200 μL of 80% ethanol, and incubate at RT for 1 min.
  i. Discard supernatant. While sample is still against magnet, incubate beads at RT for 1 min. to air-dry the beads.
  j. Remove the sample from magnet, resuspend beads in 30 μL of Elution Buffer, and incubate at RT for 5 min.
  k. Place sample against magnet, incubate until solution is clear, and transfer supernatant to a new sample tube or well of a PCR plate.
  l. Quantify sample using Qubit. Record this value.
    **Pause point:** Samples may be stored at −20°C for up to 3 days.
49. Biotin Enrichment.
  a. Mix T1 Beads very well before using, making sure that the solution is homogenous and there is nothing sticking to the bottom of the bottle.
  b. Add 12.5 μL of T1 Beads from the Arima Library Prep Box C into a well of a strip tube for each sample.
  c. Wash the T1 Beads in each tube by adding 200 μL of Binding Buffer.
  d. Mix by pipetting up and down 20 times, cap the tubes, and vortex at high speed for 5–10 seconds.
  e. Place tubes against a magnet and incubate 5 min or until solution is clear.
  f. Discard supernatant and remove the tube from magnet.
  g. Repeat steps 49.c–49.f two more times for a total of three washes.
  h. Resuspend beads in 200 μL of Binding Buffer.
  i. Transfer exactly 200 ng of size-selected DNA into a new microcentrifuge tube, PCR tube, or well of a PCR plate. If necessary, add Elution Buffer to bring sample volume to 30 μL.
    ***Note:*** Biotin enrichment and subsequent library preparation has been optimized to deliver peak performance for DNA inputs of 200 ng. Using 200 ng of DNA input has been shown to build libraries with sufficient complexity for up to 600M read-pairs of sequence data. If the amount of DNA is less than 200 ng, add in the entire amount. We have never tested less than 100 ng for biotin enrichment.
  j. Add 200 μL of washed T1 Beads in Binding Buffer, mix thoroughly by pipetting, and incubate at RT for 15 min.
  k. Place the sample against the magnet and incubate until solution is clear. Discard supernatant and remove sample from magnet.
  l. Wash beads by resuspending in 200 μL of Wash Buffer and incubate at 55°C for 2 min. Set lid temperature to 85°C.
  m. Place the sample against the magnet and incubate until solution is clear. Discard supernatant and remove sample from magnet.
  n. Wash beads by resuspending in 200 μL of Wash Buffer and incubate at 55°C for 2 min. Set lid temperature to 85°C.
  o. Place the sample against the magnet and incubate until solution is clear. Discard supernatant and remove sample from magnet.
  p. Resuspend beads in 50 μL of Deionized/Nuclease-free Water.
50. Library preparation of Enriched Ligation products.
  a. Thaw reagents and mix reagents.
    ***Note:*** Thaw ligation buffer and vortex on high to make sure the buffer is homogenous (buffer is highly viscous).
  b. Prepare Ligation master mix as shown in the table below, to allow equilibration to 20°C–25°C before use:
    ***Note:*** When preparing master mixes for library preparation of Arima Hi-C ligated material, adjust each reagent volume according to the number of reactions required, adding an additional 12% to compensate for potential pipetting losses.

**Table:** 

Reagent	Volume per reaction (μL)
Ligation Buffer	23
T4 DNA ligase	2
**Total**	**25**
  c. Keep Ligation Master Mix at 20°C–25°C for 30–45 min before use.
  d. Vortex thawed vial of End Repair-A Tailing Buffer for 15 seconds - continue vortexing until no solids are observed.
  e. Prepare End Repair/dA-Tailing master mix by combining reagents as listed in the table below, mix well and spin down.

**Table:** 

Reagent	Volume per reaction (μL)
End Repair-A Tailing Buffer	16
End Repair-A Tailing Enzyme mix	4
  f. Add 20 μL of the End Repair/dA-Tailing master mix to each sample containing 50 μL of bead-bound Hi-C library. Mix well.
  g. Program thermal cycler for End Repair and dA-Tailing using the parameters in the table below.

**Table:** 

Temperature	Time
20°C	15 min
72°C	15 min
4°C	hold
51. Adapter ligation.
  a. Once thermal cycler has reached 4°C hold step, transfer samples to ice while preparing the ligation reaction.
  b. Add 25 μL of Ligation Master Mix, to the 70 μL of bead-bound, end-repaired, and dA-tailed Hi-C library. Mix well.
  c. Add 5 μL of Adaptor Oligo Mix to each sample. Mix well.
  d. Briefly spin tubes with the bead-bound Hi-C library, Ligation master mix, and Adaptor Oligo Mix.
  e. Program the thermal cycler for the ligation step as shown below:

**Table:** 

Temperature	Time
20°C	30 min
4°C	hold
  f. After the “Ligation” program completes, remove the samples from the thermocycler and quick spin the tubes to remove any liquid from the caps. Place the sample against the magnet and incubate until solution is clear. Remove and discard supernatant.
  g. Resuspend beads in 200 μL Wash Buffer. Mix by pipetting. Incubate at 55°C for 2 min. Set the lid temperature to 85°C.
  h. Magnetize beads until the liquid is clear. Remove and discard supernatant.
  i. Resuspend the beads in 34 μL of Deionized Water and proceed immediately to Library Amplification below.
52. Amplification of Adapter-Ligated Hi-C library and Sample Indexing.
  a. Thaw and mix the Arima library reagents as shown below:

**Table:** 

Reagent	Thaw	Mix	Cap
Herculase II Fusion DNA Polymerase	Ice	Pipette	Red
5× Herculase II Buffer with dNTPs	RT	Vortex	Clear
Index Primer Pair 1-16	RT	Vortex	Foil
  b. Thaw only the index primers needed for experiment to minimize freeze-thaw cycles.
  c. Determine the unique index pair assignment for each sample using the table below as a reference:

**Table:** 

Primer pair #	P7 index forward	P5 index forward
1	CAAGGTGA	ATGGTTAG
2	TAGACCAA	CAAGGTGA
3	AGTCGCGA	TAGACCAA
4	CGGTAGAG	AGTCGCGA
5	TCAGCATC	AAGGAGCG
6	AGAAGCAA	TCAGCATC
7	GCAGGTTC	AGAAGCAA
8	AAGTGTCT	GCAGGTTC
9	CTACCGAA	AAGTGTCT
10	TAGAGCTC	CTACCGAA
11	ATGTCAAG	TAGAGCTC
12	GCATCATA	ATGTCAAG
13	GACTTGAC	GCATCATA
14	CTACAATG	GACTTGAC
15	TCTCAGCA	CTACAATG
16	AGACACAC	TCTCAGCA
  d. Prepare the appropriate volume of PCR reaction mix in the table below. Mix well.

**Table:** 

Reagent	Volume per reaction (μL)
5× Herculase II Buffer with dNTPs (clear cap)	10
Herculase II Fusion DNA Polymerase (red cap)	1
  e. Add 11 μL of the PCR reaction mixture prepared from the table above to 34 μL of Adaptor Ligated Bead Bound Hi-C Library.
  f. Add 5 μL of the appropriate, unique, Index Primer Pair to each sample.
    ***Note:*** Make sure to take note of which index was used with each sample.
    **CRITICAL:** For low-input Hi-C libraries, we highly recommend a 1:2 dilution of the adapters provided in the Arima Library kit (see problem 10).
  g. Program thermal cycle according to the settings below:

**Table:** 

Steps	Temperature	Time	Cycles
Initial Denaturation	98°C	2 min	1
Denaturation	98°C	30 sec	7–9 cycles
Annealing	60°C	30 sec	
Extension	72°C	1 min	
Final extension	72°C	5 min	1
Hold	4°C	Hold

    ***Note:*** When amplifying low-input libraries 300,000 cells, 500,000 cells or 700,000 cells we recommend using no more than 7–9, 8–10 or 10–12 cycles of amplification respectively. It is important at this point to emphasize that for the low input libraries a higher number of cycles will lead to over-amplification, which may affect the Hi-C data quality and downstream analysis (high percentage of adapter dimers, lower library complexity, etc.). For libraries starting with ≅1,000,000 mitotic cells we recommend using 12 cycles of amplification. For more details on how to avoid over amplification, please see problem 10.
53. Library purification.
  a. Add 50 μL of DNA Purification Beads to each 50 μL amplified Indexed sample. Mix well.
  b. Incubate for 5 min at 20°C–25°C.
  c. Place the sample against the magnet and incubate until solution is clear.
  d. Discard supernatant. While sample is still against magnet, add 200 μL of 80% ethanol, and incubate at RT for 1 min. Repeat this step one more time.
  e. Discard supernatant. While sample is still against magnet, incubate beads at RT for 3–5 min to air-dry the beads.
  f. Remove the sample from magnet, resuspend beads in 15 μL of Deionized/Nuclease-free Water, and incubate at RT for 5 min.
  g. Place sample against magnet and incubate until solution is clear.
  h. Remove purified and complete Hi-C library and transfer to a fresh PCR strip tube.
  i. Quantify sample using Qubit using 1 μL.
  j. Run the sample from the previous step on a gel or other platform to determine the size distribution of the Hi-C library (Figure 4).

***Note:*** Samples may be stored at −20°C for up to 6 months.

**Figure 4:**
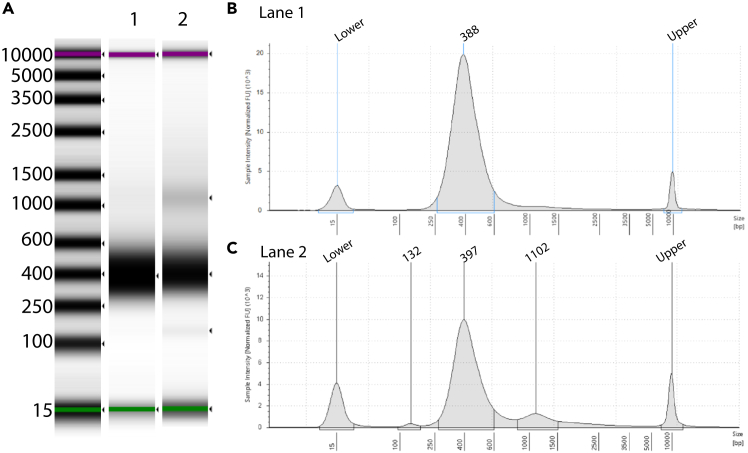
Depiction of final Hi-C library profiles in mitotic cells (A) Gel image of an optimal Hi-C library (Lane 1) and non-optimal Hi-C library (Lane 2) distribution on mitotic cells from a D5000 screen tape. The green band indicates the lower size marker added to each sample in the sample buffer. (B) Corresponding electropherogram, showing the distribution of a successful Hi-C library in mitotic cells spanning between 250–600 bp, with a peak around 400 bp. (C) Corresponding electropherogram of a non-optimal Hi-C library showing a weak peak of adapter-dimers (∼130 bp) and a stronger peak of a “PCR bubble” forming a shoulder (>1 kb band). This library profile indicates that the library amplification needs further optimization for low-input samples. For further troubleshooting on how to eliminate these bands please see problem 10. The lowermost (15 bp) and the uppermost (10,000 bp) bands represent the lower and upper DNA markers, respectively.

## Expected outcomes

FACS-based isolation of mitotic cells using this protocol produces highly pure samples of prometaphase-arrested cells (Figure 1). In our hands, this protocol can achieve about a 99% mitotically pure sample (Figures 1B and 2B). The protocol has been optimized to prevent disruption of nuclear membranes and 3D architecture (Figures 2B and 2C).

For cell types that have not been used for Hi-C in the past, we recommend first doing a pilot experiment to identify the input material that you will need as a starting material. For Hi-C that was performed in <1,000,000 mitotic cells, the proximally-ligated material ranges between 150 ng–1 mg (Figure 3). Low-input library preparation usually generates a smear that spans between 200–600 bp with the peak around 400 bp (Figure 4). For low-input libraries, “PCR bubbles” can be avoided by diluting the Illumina adapters, while a stringent size selection will help to deploy the adapter-dimers that might affect the data quality. Usually, the Hi-C sequencing depth depends on the compartments, TADs, or loop characterization needed for each project.

For this Hi-C protocol on mitotic cells, a total of 500M to 700M reads were sequenced per sample on an Illumina NovaSeq 6000 flow cell in PE100 mode. The final Hi-C libraries generated had over 60% uniquely mapped reads, and after processing with the Hi-C Pro pipeline,^12^ approximately 47% and 53% of these were identified as deduplicated valid pairs for asynchronous and mitotic cells, respectively. Alignment statistics for low input HI-C on mitotic cells when starting with 300,000, 500,000 and/or 700,000 cells are summarized in Table 1.

**Table 1:** Hi-C sequencing analysis of asynchronous versus low-input mitotic samples

Cell count	Asynchronous		Mitotic					
	400,000		300,000		500,000		700,000	
	Count	Fraction	Count	Fraction	Count	Fraction	Count	Fraction
Total	688313834	100%	549155391	100%	592732013	100%	692685127	100%
Unmapped	4562571	1%	4974550	1%	4338013	1%	5569178	1%
Uniquely Aligned	531985806	77%	399451676	73%	443910897	75%	528653608	76%
Valid Pairs	497028198	72%	372192547	68%	414941857	70%	490502255	71%
Valid Pairs (deduplicated)	326538224	47%	282489559	51%	286662098	48%	335379613	48%
Cis (<=20 kb)	82584736	12%	49372783	9%	52277592	9%	71656001	10%
Cis (>=20 kb)	205375147	30%	202834383	37%	208649815	35%	235045308	34%
Cis Total	287959883	42%	252207166	46%	260927407	44%	306701309	44%
Trans	38578341	6%	30282393	6%	25734691	4%	28678304	4%

## Limitations

Cell lines may vary in their ability to produce enough genomic DNA for low-input Hi-C library generation, and the input must be tested before beginning. Mitotic cell yield is limited to the number of cells that can be cultured and sorted to prepare the samples. If the cell line selected does not get highly enriched for prometaphase-arrested cells following nocodazole treatment, the treatment can be extended, or the number of sorting experiments can be multiplied to reach the desired number of cells. Other methods of cell cycle synchronization could also be explored, including using a double thymidine block released into nocodazole, a G2/M-arrest using RO-3306 released into nocodazole, or other cell cycle arrest agents, like S-trityl-L-cysteine (STLC).^14^^,^^15^^,^^16^

Snap-frozen cell pellets can be combined/pooled during Hi-C library preparation to meet the desired cell number. This protocol was optimized for low-input samples; however, if less than 100 ng proximally ligated material is obtained with the Arima Hi-C kit, then we recommend not proceeding with library preparation and instead bank the material until more mitotic cells can be sorted.

Sequencing depth remains one of the biggest limitations for Hi-C, with the resolution of 5–10 kb requiring hundreds of millions to billions of reads per sample, making it cost-intensive. Hi-C requires millions of cells to generate high-quality interaction maps. However, for low-input Hi-C, low complexity libraries due to the excess amount of PCR duplicates are also considered as another major limitation of this methodology, as it affects both data quality and interpretation.

## Troubleshooting

### Problem 1

No/low enrichment between asynchronous and mitotic samples (related to Step 3).

### Potential solution

Efficacy of nocodazole arrest may differ between cell lines. In our hands, we saw a 5–10 fold increase in mitotic cells following a 4-hour nocodazole treatment. Determine the percentage of cells that are arrested in prometaphase in the chosen cell line via immunofluorescence of MPM2. Below is an immunofluorescence protocol used to determine the percentage of cells.

• Synchronize cells via a nocodazole arrest.
• Seed 300,000 cells on poly-L lysine coverslips in a well of a 6-well cell culture plate.
• Grow cells to about 75% confluency and treat with DMSO or 100 ng/mL of nocodazole for 4 h.
• After incubation, aspirate cell culture media and wash coverslips with 2 mL 1× PBS.
• Fix cells with 2% PFA for 12 min at 20°C–25°C.
• Wash coverslips with 2 mL 1× PBS. Coverslips can be stored in PBS at 4°C for several months.
• Stain cells with anti-MPM2 antibody.
  ○ Remove PBS and permeabilize cells in 0.3% TritonX-100 for 10 min at 20°C–25°C.
  ○ Block cells in 0.3% BSA for 1 h.
  ○ Stain cells with anti-MPM2 antibody diluted 1:500 in blocking buffer (1% BSA) for 2 h at 20°C–25°C.
  ○ Wash cells three times in 0.1% TritonX-100.
  ○ Incubate cells with Alexa Fluor 488 goat anti-Mouse secondary antibody for 1 h at 20°C–25°C.
    - Prepare secondary antibody 1:1000 in blocking buffer (1% BSA).
• Wash cells 3× in 0.1% TritonX-100.
• Counterstain cells with Hoechst 1:2000 in PBS and mount coverslip on microscope slide.
• Image cells on a confocal imaging microscope to determine the effectiveness of the nocodazole arrest on cells.

If nocodazole treatment does not induce a sufficient or robust mitotic arrest, optimize the timing of treatment or consider using an alternative mitotic arrest treatment, such as STLC, double thymidine, or an RO-3306 block into nocodazole.^14^^,^^15^^,^^16^

### Problem 2

Not obtaining enough mitotic cells during sorting (related to Step 6).

### Potential solution

To obtain more mitotic cells, the number of cells seeded at the beginning of the experiment can be scaled up. For slow-growing or difficult-to-arrest cells, more than six cell culture dishes may need to be used to obtain enough mitotic cells. This entire protocol can be scaled up to the number of plates needed. Additionally, following sorting, snap-frozen pellets can be counted and banked. Mitotic cell isolation can be repeated until enough mitotic cells are sorted to obtain the desired cell number.

Additional components can be added to the sorting buffer to increase the output of mitotic cells without scaling up the experiment. The sorting strategy includes excluding doublet cells. To decrease the amount of doublets and prevent cell aggregation, the sorting buffer can be altered to include up to 20% BSA or up to 1 mM EDTA. Lastly, sorting at 37°C instead of 4°C may increase cell numbers; however, we have not tested the effect this may have on 3D chromatin interactions.

### Problem 3

The sorted mitotic samples are not pure and contain asynchronous cells (related to Step 6h).

### Potential solution

There are several sorting modes on the BD FACSAria III Cell Sorter. To ensure the purity of the mitotic samples, ensure the “4-way purity” mode is used during sorting. The 4-way purity mode will sort the cells at a slower rate than other modes of sorting, however it is critical to use. This mode sorts only one drop at a time, preventing potential contamination by sorting multiple drops at once. Additional contamination may occur if samples are sorted at a flow rate above 1,500–3,000 events/second. Keep the flow rate low and dilute samples as needed to achieve this.

### Problem 4

Asynchronous cells 3D structure disrupted after sorting (related to Step 4 and/or Step 6).

### Potential solution

To preserve the 3D nuclear structure of cells during and after sorting, several precautions should be taken. First, cells should be fixed prior to sorting using 2% PFA for 10–15 minutes, or up to 3% PFA for particularly fragile cell types. For highly sensitive samples, a dual crosslinking approach—such as pre-treatment with DSG or EGS, followed by PFA—can offer enhanced stabilization of chromatin architecture. During sorting, it is important to use low-pressure settings, such as a larger nozzle and reduced psi, and to keep cells chilled throughout the procedure to minimize mechanical stress. We recommend that upon sorting, cells should be collected into a BSA-containing buffer to help them during recovery; for ultra-sensitive cell types, a buffer containing up to 30% BSA in 1× PBS can be used. Finally, post-sort handling should be fast as possible, while avoiding prolonged room temperature (20°C–25°C) exposure, as it can contribute to the loss of 3D structure.

### Problem 5

After centrifugation of flow cytometry samples, the cell pellets are not visible or very small (related to Step 7).

### Potential solution

If a pellet is not visible, keep the supernatant and increase the concentration of BSA in the sample to a maximum of 0.3% BSA. Mix the sample thoroughly and centrifuge again. Recounting the cells following sorting can help determine size of pellet that should be expected. Increase centrifugation speed up to 3000 × *g*.

### Problem 6

Insufficient input chromatin (related to Step 26).

### Potential solution

We highly recommend recounting the cells after sorting. Cells can be lost during the sorting process due to the mechanical force. If you are setting up a small-scale experiment or have extremely low cell numbers, this is something you should take into consideration. If your sorted cells are not enough to proceed with Hi-C, then we recommend freezing them and starting a new batch of sorting and pooling the pellets together for your Hi-C. Pooling sorted and fixed cells from different batches has been working in our hands successfully.

### Problem 7

Partially digested/ligated chromatin (related to Step 35).

### Potential solution

Partial ligation events observed during Hi-C library preparation are often the result of partial chromatin digestion, which is usually caused by inefficient cell lysis. Partial lysis can limit affect enzymes reaching the chromatin, particularly in larger cell types such as immune cell types with fluctuating nuclear-to-cytoplasmic ratio (N/C): such as activated T cells (TH), Thioglycolate-elicited peritoneal macrophages (TEPMs) or megakaryocytes, where the nuclear membrane may be more resistant to standard lysis conditions especially upon cell activation, during an immune response. To improve digestion efficiency in these large cells, we recommend increasing the lysis incubation time. In our experience, extending the lysis step to 20–25 minutes (from the standard 15 minutes) significantly enhances nuclear accessibility, leading to more complete digestion and reducing the risk of partial ligation downstream for the COLO320-DM cells. Prior to initiating a full Hi-C experiment, we strongly recommend performing a pilot enzymatic digestion test to ensure optimal digestion conditions.

### Problem 8

Inefficient/suboptimal sonication of proximally Hi-C ligated chromatin (related to Step 47).

### Potential solution

Suboptimal fragmentation of proximally ligated chromatin can affect library quality and complexity. Inefficient sonication often leads to either under- or over-fragmentation, both of which can negatively impact downstream library preparation and sequencing results. This issue is frequently related to variability in sonication performance across different instruments and protocols. Factors such as sonication tube type, sample volume, DNA concentration, and instrument performance all contribute to the efficiency and reproducibility of DNA shearing. We highly recommend testing your equipment for DNA shearing. This allows you to calibrate conditions that are optimal for your specific setup and input amount. Sonication conditions vary considerably between instruments (e.g., Covaris vs. Bioruptor). In our hands, the Covaris E220 has proven to be highly effective for low-input Hi-C proximally-ligated material when used with the appropriate consumables and settings. When using Covaris microTUBE AFA Fiber Pre-Slit Snap-Cap 130 μL, ensure the final volume is precisely 100 μL for optimal shearing efficiency. Ideally, 200–250 ng of proximally ligated DNA (measured by Qubit or equivalent fluorometric method). Estimating DNA concentration before sonication allows you to avoid overly aggressive shearing conditions that could fragment DNA beyond the desired size range. Since ligation efficiency may vary between different cell lines, having an electropherogram analysis will help us to further determine the sonication conditions (Figure 3A). This is particularly critical for low-input samples, where over-sonication can significantly reduce yield and compromise complexity.

### Problem 9

Reduced number of mapped 3D interactions.

### Potential solution

This issue may stem from disrupted 3D nuclear architecture or incomplete fixation of the cells. In either case, we recommend checking the 3D structure using DAPI staining or immunofluorescence staining for nuclear proteins, such as Lamin or Nuclear Pore Complexes (NPCs). Another factor that could lead to reduced chromatin interactions is the partial fixation. In our experience, increasing the paraformaldehyde (PFA) concentration can help better stabilize chromatin interactions. We have successfully used PFA concentrations up to 3% for Hi-C without compromising enzymatic digestion and the rest of the steps. If increasing the PFA concentration is not a good option for your samples, then we recommend keeping the 2% PFA and increasing the fixation time from 10 minutes to 15 minutes. For any fragile or sensitive cell types where mechanical stress during the protocol may affect their cellular structure, we also recommend using a secondary crosslinker such as EGS or DSG to reinforce chromatin structure. Lastly, we recommend adjusting centrifugation conditions for sensitive cells to minimize mechanical stress. However, note that reducing centrifugation force can increase the risk of cell loss, so adjustments should be carefully optimized.

### Problem 10

Observation of a ∼1.2 kb band and/or adapter-dimers in the library prep (related to Step 52).

### Potential solution

Since this is a low-input Hi-C (typically ranging from 300,000 to 500,000 cells) the amount of material used for each library is going to be reduced when compared with standard-input Hi-C protocols. As a result, the quantity of DNA available for library construction is proportionally reduced. When standard concentrations of Illumina adapters that are provided with the library kit are used under these conditions, the excess amount of adapters relative to DNA can lead to undesirable side effects during PCR amplification, generating the formation of adapter dimers or “bubble artifacts” in the final library. These “PCR bubbles” appear as high-molecular-weight bands around 1–1.2 kb during TapeStation analysis (Figures 4A and 4C). To detect the presence of a “PCR bubble” in your library, we recommend using the D5000 Agilent tapes for the visualization, as this band won’t be visible in the D1000 Agilent tapes. Adapter dimers, a band observed at 130 bp, may also affect the library data quality. To eliminate these issues, a maximum of 7–9 cycles of library amplification should be used as overamplification will affect data quality. For low-input Hi-C, we also suggest diluting the adapters proportionally. In our lab, when starting with approximately 300,000 cells, a 1:2 dilution of Illumina adapters led to better quality libraries. However, it’s important to note that this strategy is empirical and may need to be optimized depending on sample specificity, as well as the cell numbers used initially as input for each experiment. We highly recommend conducting a small pilot test to evaluate the effect of adapter dilution on your specific cell type.

## Resource availability

### Lead contact

Further information and requests for resources and reagents should be directed to and will be fulfilled by the lead contact, John Maciejowski (maciejoj@mskcc.org).

### Technical contact

Technical questions on sorting and isolating mitotic cells should be directed to and will be answered by the technical contacts, Ashley Nichols (nichola3@mskcc.org). Technical questions related to Hi-C should be directed to and will be answered by the technical contact, Eralda Salataj (salatae1@mskcc.org).

### Materials availability

This study did not generate new unique reagents.

### Data and code availability

This study did not generate or analyze new datasets or code.

## Acknowledgments

Work in J.M.’s laboratory is supported by the NCI (R37CA261183, R01CA270102, R01CA304441, and P30CA008748), the Pershing Square Sohn Cancer Research Alliance, the Frank A. Howard Scholars Program, the Mary Kay Ash Foundation, and the Experimental Therapeutics Centers at MSKCC. This material is based upon work supported by the National Science Foundation Graduate Research Fellowship under grant no. 2234691 (A.N.).

## Author contributions

Conceptualization, A.N., E.S., and J.M.; methodology, A.N. and E.S.; investigation, A.N. and E.S.; formal analysis, A.N., Y.C., and E.S.; writing – original draft, A.N. and E.S.; writing – review and editing, all authors; visualization, A.N. and E.S.; funding acquisition, A.N. and J.M.; supervision, P.-J.H., R.K., and J.M.

## Declaration of interests

R.K. is a co-founder of and consultant for Econic Biosciences.
